# Dynamics of Transgenic *Enterobacter cloacae* Expressing Green Fluorescent Protein Defensin (GFP-D) in *Anopheles stephensi* Under Laboratory Condition

**Published:** 2017-12-30

**Authors:** Hossein Dehghan, Mohammad Ali Oshaghi, Seyed Hassan Moosa-Kazemi, Bagher Yakhchali, Hassan Vatandoost, Naseh Maleki-Ravasan, Yavar Rassi, Habib Mohammadzadeh, Mohammad Reza Abai, Fatemeh Mohtarami

**Affiliations:** 1 Department of Medical Entomology and Vector Control, School of Public Health, Tehran University of Medical Sciences, Tehran, Iran; 2 Department Industrial and of Environmental Biotechnology, National Institute of Genetic Engineering and Biotechnology, Tehran, Iran; 3 Department of Chemical Pollutants and Pesticides, Institute for Environmental Research, Tehran University of Medical Sciences, Tehran, Iran; 4 Malaria and Vector Research Group, Biotechnology Research Center, Pasteur Institute of Iran, Tehran, Iran; 5 Department of Parasitology and Mycology, Faculty of Medicine, Urmia University of Medical Sciences, Urmia, Iran

**Keywords:** Bacterial dynamic, *Enterobacter cloacae*, *Anopheles stephensi*, Paratransgenesis

## Abstract

**Background:**

*Enterobacter cloacae* bacterium is a known symbiont of the most *Anopheles* gut microflora and nominated as a good candidate for paratransgenic control of malaria. However, the population dynamics of this bacterium within *An. stephensi* and its introduction methods to the mosquitoes have not yet been explored.

**Methods:**

*Enterobacter cloacae* subsp. *dissolvens* expressing green fluorescent protein and defensin (GFP-D) was used to study transstadial transmission and the course of time, larval habitat, sugar, and blood meal on dynamics of the bacterium in the mosquito life stages in the laboratory condition. The bacterial quantities were measured by plating samples and counting GFP expressing colonies on the Tet-BHI agar medium.

**Results:**

The *E. cloacae* population remained stable in sugar bait at least for eleven days whereas it was lowered in the insectary larval habitat where the bacteria inadequately recycled. The bacterium was weakly transmitted transstadially from larval to adult stage. The bacterial populations increased smoothly and then dramatically in the guts of *An. stephensi* following sugar and blood meal respectively followed by a gradual reduction over the time.

**Conclusion:**

*Enterobacter cloacae* was highly stable in sugar bait and increased tremendously in the gut of female adult *An. stephensi* within 24h post blood meal. Sugar bait stations can be used for introduction of the transgenic bacteria in a paratransgenic approach. It is recommended to evaluate the attraction of sugar bait in combination with attractive kairomones as well as its stability and survival rate in the semi-field or field conditions.

## Introduction

Malaria is a mosquito borne disease considered as an important threat to public health in tropical and semi-tropical areas of the world. Although Iran currently is in elimination phase and malaria cases are significantly reduced, the disease is still considered a serious health concern, mostly in the south and southeast corner of the country (1–3).

In the southern regions of the country there are six *Anopheles* mosquito vectors including *Anopheles stephensi*, *An. culicifacies* s.l, *An. dthali*, *An. fluviatilis* s.l, *An. superpictus* s.l. and *An. pulcherrimus* (4–12). However, *An. stephensi* has been considered the main malaria vectors in Iran (13–15). This species has shown a wide range of susceptibility/resistant to various insecticides in Iran (16–20). Currently, this species is resistant to lambda cyhalothrin and cyfluthrin and susceptible to etofenprox, and permethrin and candidate of resistant to deltamethrin in the country (21). *Anopheles stephensi* is one of the most important malaria vectors in Middle East and Indian subcontinent regions and its resistance to organochlorides, organophosphates, carbamates and pyrethroids insecticides have been widely reported in these regions (22–24).

Emergence of insecticide resistant mosquitoes plus the emergence of drug-resistant in the parasites highlights the needs for alternative strategies for sustainable malaria control. As an alternative to chemical insecticides, paratransgenesis depends on engineered symbiotic microorganisms particularly bacteria of malaria vectors to supply molecules that can kill *Plasmodium* inside the mosquito gut and or inhibit pathogen transmission (25, 26). Transmission of the *Plasmodium* parasite is strongly dependent on completion of the parasite life cycle in the mosquito vector since entering to midgut, across the peritrophic membrane (PM), midgut epithelium, salivary glands and transmitted through saliva to new host. The most bottleneck during the development of *Plasmodium* parasite occurs in ookinete stage. It could be considered as the main target for control of parasite in mosquito vectors (27–31).

Some known factors involved in creating parasite bottleneck, including gut digestive enzymes, intestinal microbial flora, and the mosquito’s immune response. Microflora performs vital role in preventing the development of pathogens. This effect exerts directly by proliferation of bacteria after blood meal simultaneously with ookinete stage of *Plasmodium*. Besides, indirect effects on the survival of *Plasmodium* parasite are applied by inducing the expression of anti-microbial genes against the bacteria (32–38). Accordingly, bacterial symbionts are genetically modified to express toxic peptides against pathogens, can be considered as an alternative approach for disease control (39). This strategy, commonly named paratransgenesis (40), requires several steps of research on the biology of vectors and vector symbionts and its evaluation in the laboratory and field conditions (41–43).

The midgut bacterial flora of wild-caught mosquitoes is very dynamic and significant fluctuations depending on the stage of life, nutrients and the physiological age (31, 44). Population structure of symbiotic bacteria is considerably changed post blood meal and gram-negative bacteria will be dominant and could survive in harsh condition of midgut with digestive enzymes (31). There are scattered studies on insect gut microflora (15, 45, 46 and references herein) and still remained some questions in relation to species composition, stability as well as their acquisition of microbiota (47–49). Punpuni et al. (1996) (33) reported at least nine species of cultivable midgut bacteria with varied composition of *An. stephensi*, *An. gambiae* and *An. albimanus*. In the same study, variety composition of midgut bacteria flora was found in *An. gambiae* and *An. funestus* and some *Anopheles* mosquitoes (49–59).

A few of mosquito microflora able to pass from larvae to adult stages because of the differences between the larval (Aquatic) and adults (Terrestrial) habitats (41, 44). Some bacteria are able to colonize in the malpighian tubules and transstadially pass from larvae to adult and presumably remain for long duration in the female gut (15). Therefore, such symbiotic bacteria added to the diet of adult mosquitoes (60). *Enterobacter cloacae* bacterium is a species of gram-negative, facultative anaerobic, rod-shaped bacteria belonging to Gammaproteobacteria and Enterobacteriaceae family. The bacteria species limited the development of *Plasmodium berghei* and *P. falciparum* by stimulate the immune system of *An. stephensi* and increases the expression of immune responses compounds such as serine protease inhibitors (SRPN6) (31). *Enterobacter cloacae* bacterium was found as the microflora of *An. stephensi* (57). Gonzalez-Ceron et al. (51) reported *E. cloacae* restricted the *P. vivax* development in midgut of *An. albimanus*. The bacterium also was reported from *Culex tarsalis* (61), *Psorophora columbiae* (62), *Aedes triseriatus* (62) and *Ae. albopictus* and *Ae. aegypti* (63). Due to the ability of *E. cloacae* to direct and indirect control of *Plasmodium* parasites, these bacteria could be introduced as a candidate for paratransgenesis approach against the malaria parasite. Maleki-Ravasan et al. (46) suggested *E. cloacae dissolvens* as a candidate for paratransgenesis approach to control of *Leishmania* transmission in the sand flies vectors. They genetically modified the bacterium to produce defensin as a *Plasmodium* killing effector protein. Defensins are small cysteine-rich cationic proteins and found in plants, vertebrates and invertebrates. They are active against fungi, bacteria, and many viruses. To use recombinant bacteria in practice, however, it is required a better understanding of the bacteria dynamics in mosquitoes and its delivery systems to vectors.

In this study, we evaluated the dynamics of *E. cloacae dissolvens* expressing green fluorescent and defensin proteins (GFP-D) in midgut of *An. stephensi* life stages as well as in larval habitats and sugar bait used as two delivery systems for the mosquito in the laboratory condition.

## Materials and Methods

### The mosquitoes

*Anopheles stephensi*, Beech strain originally collected from Pakistan as an additional type form ie SDA500 strain originating was provided in 2005 by Professor P.F. Billingsley, Sanaria, Inc (64). Breeding of the mosquitoes carried out in 27±1 °C and 60±10% relative humidity with photoperiodic period of 12h. Adult mosquitoes were kept in 30×30×30 cages. Mosquito feeding was carried out using fructose 5% and guinea pigs twice a week. *Anopheles* gravid, laying eggs in earthenware bowl containing decolorized water. The eggs were slowly transferred to tray having 1500ml of decolorized water before hatching. The fish food and a piece of leaf lettuce used as specific diet for *Anopheles* larvae.

### The bacteria

*Enterobacter cloacae* subsp. *dissolvens* was isolated by sampling microflora of *Phlebotomus papatasi* in the field of zoonotic cutaneous leishmaniasis in Isfahan, central Iran, 2013–2014 (46). The manipulated strain of *E. cloacae* is carrying plasmid expressing difensin and GFP proteins and a gene resistant to tetracycline (Tet-gene). This strain known as *Enterobacter cloacae*-GFP-Difensin (*E. cloacae*-GFP-D) maintained in the School of Public Health, Tehran University of Medical Sciences. The bacterium was grown in Brain Heart Infusion (BHI) Broth culture until stationary phase, as determined by spectroscopic optical density (OD) measurements at 600nm. The bacteria were prepared by growing to an OD 600 of = 1 in Tet-BHI broth medium. Several dilutions of the OD 600= 1 were prepared covering a wide region of optical density from 0.1 to 1 and plated onto Tet-BHI agar for viable cell determination. The plates were incubated for 13h at 37 °C before counting the number of colony forming units (CFU). The gradients of the calibration curves showed that OD 600nm of 1.0 was corresponding to approximately 1×10^9^ CFU per ml BHI broth medium.

### Dynamics of *Enterobacter cloacae*-GFP-D Corncob-bacteria formulation (CCF) and sugar bait-bacteria

**Fig. 1.**
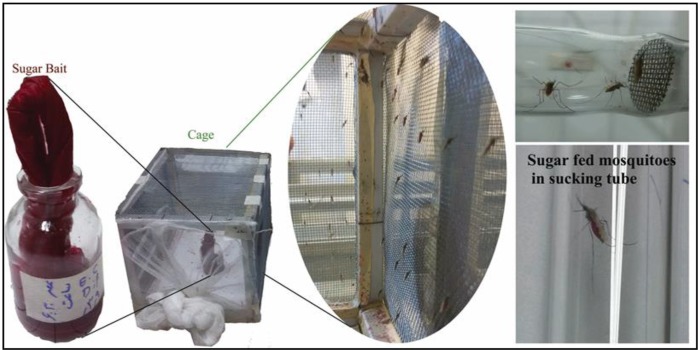
Sugar bait containing 10^9^ bacteria cell/mL (CFUs) fructose 5% and 2.5% food dyes used to introduce the *Enterobacter cloacae*-GFP-Defensin bacteria to mosquitoes via sugar feeding. The dye made abdomen reddish and visually distinguishable.

The method of Arshad et al. (65) was used to prepare corncob formulation (CCF) of *E. cloacae*-GFP-D and used as the bacterium-floating carrier in larval tray. The corncobs were autoclaved followed by grinding to small particles less than 0.5mm in diameter. The medium containing *E. cloacae* centrifuged and the precipitated cells washed and suspend in 100μL PBS. The bacteria-PBS buffer was added on corncob and dried at room temperature. Almost 100μL of bacteria-PBS suspension was used for 0.1g grind corncob. Almost 0.1g of the dried CCF containing 5×10^9^ bacterial cells was used for one litter of larval tray water in insectary condition. Sugar bait-bacteria were prepared by using 10^9^ the bacterial cell per 1ml of fructose 5% and 2.5% red food dyes (Fig. 1) according to the method previously described by Wang et al. (66).

### Introduction *Enterobacter cloacae*-GFP-D to mosquito larval habitats

About 200–300 *An. stephensi* eggs were transferred to tray containing sterile water and then the hatched larvae were transferred randomly to the test and control trays. The larvae were fed either on intact corncob and a piece of leaf lettuce in control tray or CCF and a piece of leaf lettuce in test tray. The number of released bacteria in test tray was 10^9^ bacterial cells per liter of sterile water. The CCF was added following emergence of the first (L-I) and fourth (L-IV) instar larvae in test tray. Transstadial and dynamics of the bacteria were investigated by sampling of water and mosquitoes at larvae and adult stages.

### Dynamics of *Enterobacter cloacae*-GFP-D in larval habitat

To test the course of time on proliferation and stability of *E. cloacae*-GFP-D in larval habitats of mosquitoes, water sampling was carried out daily from the test trays. For each sample, 10ml of the water was centrifuged at 13000 RPM for 10min followed by removing supernatant; the pellet was mixed with 1mL PBS buffer and vortexed. Finally, serial dilution of the bacterial suspension was prepared and 100μL of the proper dilution cultured in Tet-BHI agar. The number of colonies (colony forming units: CFUs) was counted under a fluorescent microscope and number of the *E. cloacae* bacteria in one ml of test tray water was measured.

### Bacterium transstadial transmission

**Fig. 2.**
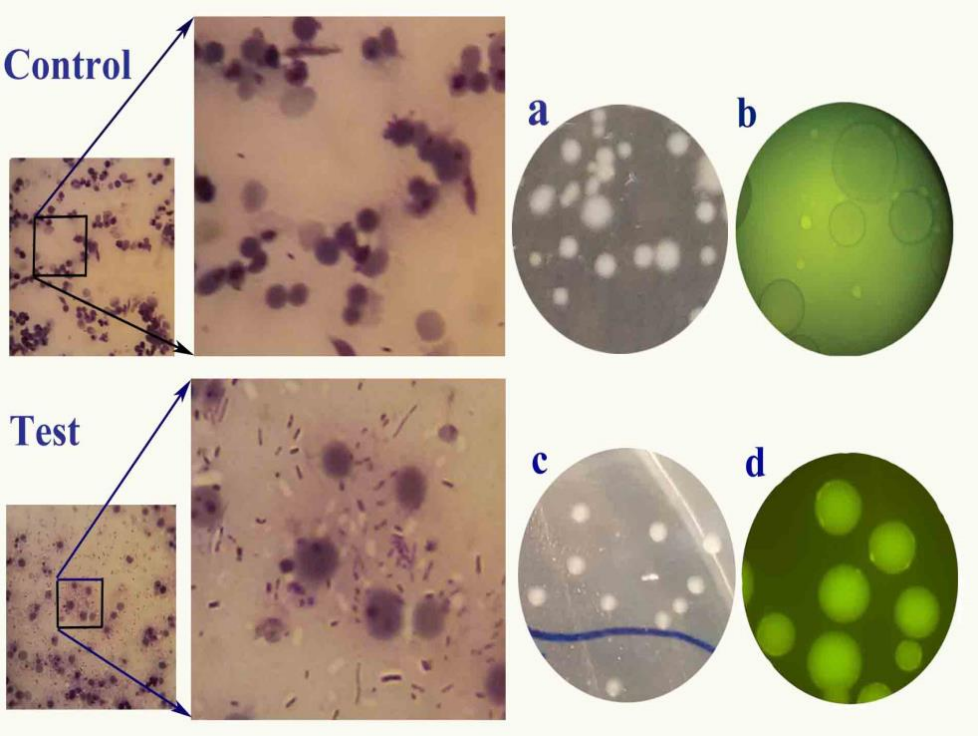
The presence of *Enterobacter cloacae*-GFP-Defensin in dissected *Anopheles stephensi* midgut (squares) and BHI agar plates (circles), the bacteria with/out expressing green fluorescent protein in BHI agar plate under non-fluorescent (a and c) and fluorescent (b and d) microscope

To test the transstadial transmission of *E. cloacae*-GFP-D bacteria from larvae to adult stage of the mosquitoes we followed the method previously described by Lindh et al. (48). On brief, 5×10^6^ bacteria as CCF per ml sterile water were released in larval breeding trays. Larvae were kept under laboratory condition until pupae stage. Fresh pupae were washed twice in sterile water and transferred to a new tray with 1000mL sterile water until adults emerged. After eclosion, the adult body surface of some specimens was sterilized in 70% alcohol, then the midgut dissected, homogenized in PBS, and cultivated in Tet-BHI agar/broth for 13h in 37 °C. However, some adults were transferred to 15×15×30cm cage and fed on sterile sugar solution during 24h. The female mosquitoes were blood fed on BALB/c mice and midgut microflora was determined by described method. The number of colonies (CFUs) was counted under a fluorescent microscope for all experiments (Fig. 2).

### Dynamics of *Enterobacter cloacae*-GFP-D in *An. stephensi* larvae

In each sample, five larvae were selected and their surface bodies were sterilized by alcohol. Briefly, the larvae were transferred to microtubes containing 500μL sterile water and kept on ice for a few minutes until the larvae were numb, then 500μL of 70% cold ethanol (−5 °C) was added to the microtube after removing water and kept on ice about 5 min. The alcohol was removed and the larvae were washed twice with PBS buffer (4 °C). Finally, the total body of sterilized larvae homogenized in PBS buffer and 100ml of homogenized solution was cultured in Tet-BHI agar plates. Alternatively, the larvae were dissected and their guts were homogenized in PBS buffer, cultured in Tet-BHI agar plates, and CFUs were counted as above. Simultaneously, survival rate of *An. stephensi* larvae in the test and control (corncob contaminated with/out *E. cloacae*-GFP-D) trays were investigated.

### Dynamics of *Enterobacter cloacae*-GFP-D in *Anopheles stephensi* adult gut

In order to study population dynamics of *E. cloacae*-GFP-D in *An. stephensi* adult, the 3–5d old female mosquitoes were transferred to 15×15×30cm cage and fed on sugar bait containing 10^9^ the bacterial cell/mL fructose 5%, and 2.5% food dyes (red) according to the method previously described by Wang et al. (66) (Fig. 1). To count the bacteria populations (CFUs) in the adult guts, the female mosquitoes were numbed and immersed in ethanol 70% for 5–7min, placed on glass slides for 3–5min, and then transferred to microtube and homogenized in PBS buffer. Finally, 100μL of homogenized suspension samples was cultured in Tet-BHI agar plates. In an alternative method, after body surface sterilization, mosquito midguts were dissected and homogenized in PBS buffer. To evaluate course of time and sugar/blood meals on the CFUs of the bacterium, two separate experiments were designed. In the first experiment, 3–5d old female mosquitoes were fed on sugar bait, a subset of specimens was tested for the number of the bacteria immediately (1 hour) post sugar feeding. Then the sugar bait was removed from the cage for 7h and again the bacteria population was tested in a subset of females. Eight hours post sugar feeding, a blood meal was offered to the mosquitoes and then a subset of mosquito guts was tested for the bacteria at 12, 18, 24, 36, 48, 96, and 144h post blood meal. After day-6 (144h), a second blood meal was offered to the flies, and CFUs were counted for the guts 24 and 36h post second blood meal.

In the second experiment, 3–4d old female mosquitoes were fed on sugar bait, a subset of specimens was tested for a number of the bacteria immediately (1 hour) post feeding. Then the sugar bait removed from the cage for 7h and again the bacteria populations were tested in a subset of females. Again, eight hours after first sugar meal, second sugar meal was offered to the mosquitoes and the CFUs were measured for the guts 24, 48, and 72h post second sugar meal. Then a blood meal was offered and a subset of mosquito guts was tested for the bacteria CFUs at 24 and 48h post blood meal. In both experiments, 3–4h in advance of offering blood meal, sugar bait containing the bacteria were removed from the cage, and after blood meal, the mosquitoes were kept on sterile cotton pad soaked with 5% fructose.

### Stability of *Enterobacter cloacae*-GFP-D in sugar bait

Colony-forming units of the bacteria in sugar bait containing 10^9^ bacteria cell/mL fructose 5% and 2.5% red food dyes was determined by daily sampling during eleven d. Sampling was carried out as follow, by pressing the cotton pad, 10μl of the above suspension was collected and added to 990μl of sterile PBS buffer and prepared serial dilutions. About 100μl of the final solution was cultured in Tet-BHI agar plates. After counting the colonies, number of bacteria per ml of bait was estimated.

### Statistics and analytical procedure

Where it was necessary, the concentrations of CFUs were indicated using logarithmic notation, where the value was shown is the base 10 logarithm of the concentration. Bacteria relative abundance was calculated separately for each treatment. The average percentage of lifespan and larval death rate were presented. A significant difference in the bacteria relative abundance between samples was analyzed using the Mann–Whitney test. Multiple-sample comparisons were analyzed using the non-parametric Kruskal–Wallis test, and medians were compared using Dunn’s test. GraphPad Prism version 5.00 for Windows (GraphPad Software) was used for all statistics. P< 0.05 was considered statistically significant.

## Results

### Corncob formulation (CCF) stability

The most attractive features of corncob were considered as lightness and floating on the water surface. When this formulation supplied on mosquito breeding place, *Anopheles* larvae attracted to and fed on corncob particles. Approximately, 90–95% and 50–60% of CCF particles remained floated on the tray water after 24 and 48h of their releasement, respectively.

### Effect of *Enterobacter cloacae*-GFP-D on survival of *Anopheles stephensi* larvae

**Fig. 3.**
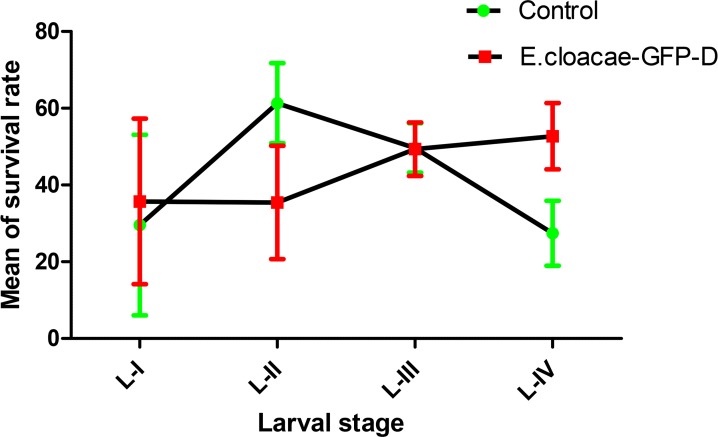
Mean of survival rate of *Anopheles stephensi* larvae in test groups (sterile water+corncob-*Enterobacter cloacae*-GFP-Defensin) and control (sterile water+corncob) throughout larval development stages (14d) in insectary condition. The bars represent standard error of the mean

**Fig. 4.**
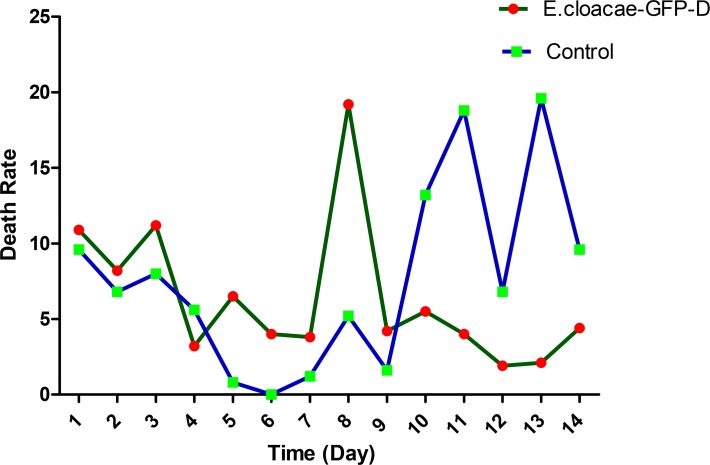
Mortality rate of aquatic stages of *Anopheles stephensi* in test (sterile water+ CCF: corncob-*Enterobacter cloacae*-GFP-Defensin) and control (sterile water+corncob) tray throughout 14d in insectary condition.

The survival rate of *An. stephensi* larvae in the test tray containing *E. cloacae*-GFP-D significantly was more than the control group at late larval stage. Vis-versa results of this experiment showed that death rate of control were significantly lower than the test group at larval stage II (Fig. 3, 4). Peak mortality in test groups occurred about days 8–9, whereas it happened about days 10–14 in control group (Fig. 4).

### Effect of *Enterobacter cloacae*-GFP-D on development rate of *Anopheles stephensi* larvae

Development of *An. stephensi* larvae took longer time when kept in sterile water or in water containing *E. cloacae*-GFP-D in comparison with the ones kept in non-sterile water. On average adult, mosquitoes appeared 8–10d from the time of egg hatching when the larvae had kept in non-sterile water whereas it took 14–18d when the larvae bred in sterile water or water containing *E. cloacae*-GFP-D. Both test and control groups were supplied by a piece of lettuce and corncob.

### Dynamics of the bacteria in the larval stages and habitat of *Anopheles stephensi*

**Fig. 5.**
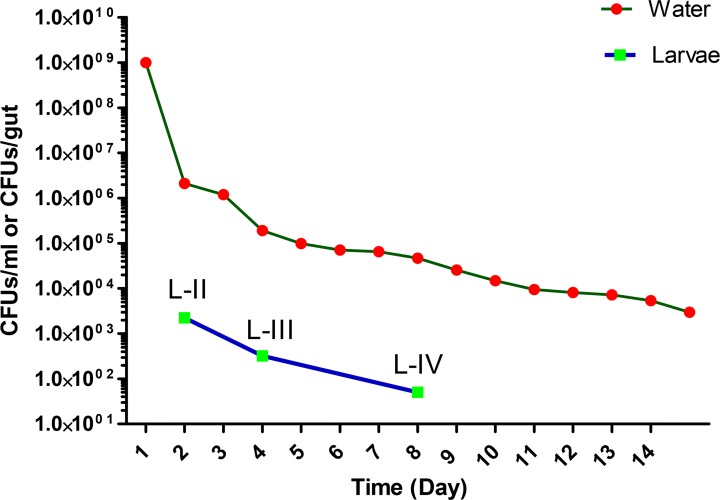
Trend of *Enterobacter cloacae*-GFP-Defensin in the *Anopheles stephensi* larval habitat water (above) and guts (down) in insectary condition. L-II, L-III, and L-IV represent larval stage II, III, and IV respectively

Population dynamics of *E. cloacae*-GFP-D had a descendant trend in the larval habitat indicating a weak recycling in water at insectary condition. The population of *E. cloacae*-GFP-D in the water decreased from 2×10^7^ CFUs/ml at day-1 to 2960 CFUs/mL at the end of day-14 (Fig. 5). Trend of the bacteria in the guts of *An. stephensi* larvae also declined significantly from about 12000 CFUs in the second instar larvae (L-II) to less than 100 CFUs in the fourth instar larvae (L-IV) which is corresponding to the decreasing trend of the bacteria in the larval habitat water (Fig. 5).

### Transstadial transmission

*Enterobacter cloacae*-GFP-D can survive and flourish in the guts of *An. stephensi* larvae but inadequately transmit transstadially from larvae to adult stage. The rate of bacteria positive in larval guts at late instars was more than 75% with a range of 7 to 756 CFUs per gut. However, most of the bacteria were removed from midgut in pupal stage due to histolysis and histogenesis phenomena. None of the pupae were positive for the bacteria and there were only 2–3 adult specimens (8–12%) were positive for *E. cloacae* subsp*. dissolvens* bacteria indicating very low transstadial transmission (Table 1). There were only a few (n= 4) bacteria in the newly emerged adult mosquito. The number of bacteria increased dramatically following a blood meal and reached to 10000 CFUs (Table 1).

**Table 1.** Details of transstadial transmission of *Enterobacter cloacae*-GFP-D in *Anopheles stephensi* in insectary condition. Numbers refer to the guts harbor the bacteria out of 25 specimens.

**Life stages**	**Tet-BHI broth**	**Tet-BHI agar (CFUs/gut)**
**Larvae IV**	19	17 (7–756)
**Pupae**	0	0
**Adult Blood Fed**	2	1 (10,000)
**Adult Unfed**	1	1 (4)

### Course of time and blood meal on *Enterobacter cloacae*-GFP-D in adult *Anopheles stephensi* midguts

**Fig. 6.**
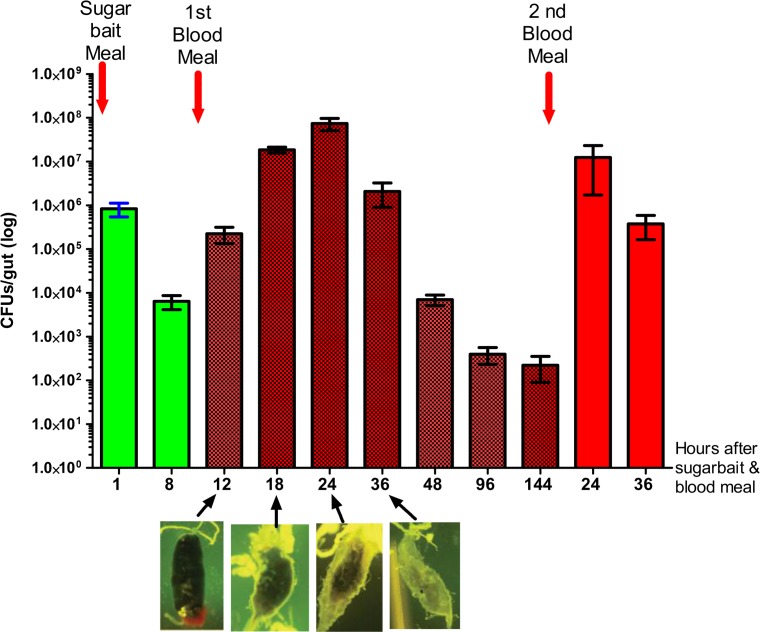
Dynamics of *Enterobacter cloacae*-GFP-Defensin populations in the midgut of adult *Anopheles stephensi* fed on sugar bait containing 10^9^ the bacteria cell/mL fructose 5%. Midgut status 12, 18, 24, and 36h post blood meal is shown underneath. The bars represent standard error of the mean

We tested the effect of blood meal and course of time on loads of the bacteria in adult mosquitoes. Number of the bacteria in the adult mosquito midgut fed on sugar bait containing *E. cloacae*-GFP-D was on average one million per mosquito gut one-hour post sugar bait feeding. However, populations of the bacteria decreased about 100 folds after seven hours fasting (Fig. 6). Loads of bacteria were dramatically increased about 73000 folds on average and maximized up to 155 million (about 73000000 on mean) CFUs 24h post blood meal. The bacteria populations were then declined gradually and reached to about 100 CFUs/gut 144h after (6d) blood meal intake. This trend, i.e. increase, and decrement of the bacteria populations happened again following second blood meal with a slightly lower rate than first blood meal (Fig. 6).

**Fig. 7.**
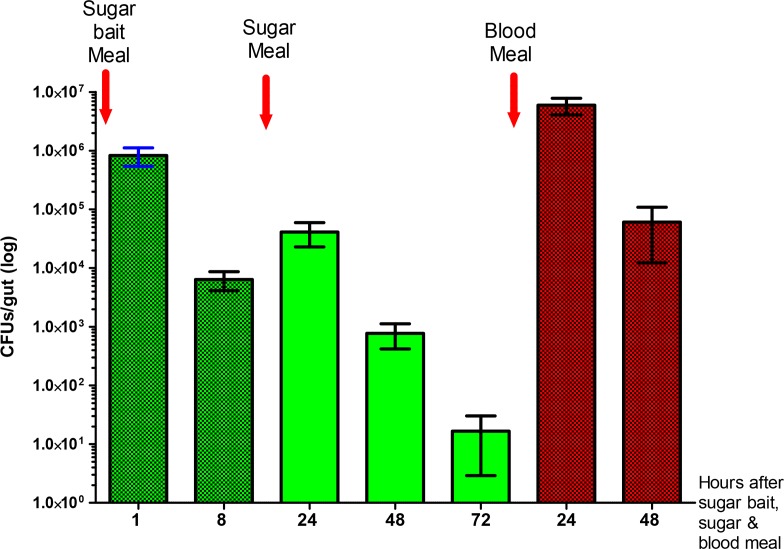
Course of time, sugar, and blood meal on the dynamics of *Enterobacter cloacae*-GFP-Defensin populations in the midgut of adult *Anopheles stephensi.* Mosquitoes were fed on sugar bait containing 10^9^ CFUs/mL fructose 5%, then kept starve for 8h, and then fed on sugar (cotton pad) for three days continuously and on blood meal after 72h. The bars represent standard error of the mean

To test the effect of only sugar meal (fructose 5%) on the bacteria population, in a separate experiment, the mosquitoes were fed on sugar bait containing *E. cloacae*-GFP-D and then starved for 8h. Then they were fed on fructose 5% with no bacterai using cotton pad and the load of bacteria was counted daily for three following days. After 24h following normal sugar meal, the bacteria population raises up about 10 folds (10000 to 99000 CFUs per gut) and again by course of time, the bacteria population decreased again (Fig. 7). The number of bacteria dropped about 1000 folds and reached to about 10 CFUs/gut after 72h (3d) (Fig. 7). A blood meal intake following three days causes tremendous (140000 folds) uprise in the number of adult guts bacteria but lower than the previous experiment (14 million versus 155 million).

### Dynamics of *Enterobacter cloacae*-GFP-D in sugar bait

**Fig. 8.**
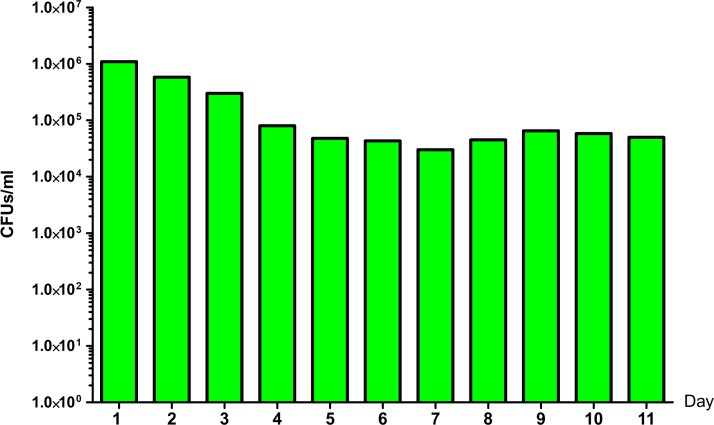
Trend of *Enterobacter cloacae*-GFP-Defensin population in the sugar bait solution containing 10^9^ CFUs/mL fructose 5% at the beginning of experiment (day zero) in insectary condition throughout 11d.

In order to determine the course of time on survival of *E. cloacae*-GFP-D in suspended sugar bait, trend of *E. cloacae*-GFP-D populations was investigated in the sugar solution of cotton pad. The number of bacteria was decreased 10 folds (from 1 million to 100000 CFU/ml) throughout 11d in the insectary condition (Fig. 8).

## Discussion

We investigated transstadial transmission and dynamics of *E. cloacae*-GFP-D within an *Anopheles stephensi* mosquito colony with the focus on larval habitat and sugar bait as two potential resources or delivery systems for introduction of the transgenic bacteria to mosquitoes. Our observations revealed that the symbiont *E. cloacae*-GFP-D strain was not able to colonize well in larvae midgut or water habitats of *An. stephensi* in laboratory rearing condition. The number of bacteria reduced progressively in both water and larvae midgut. Besides, number of the bacteria transferred transstadially was very low (4%). In this experiment, the bacteria were administrated via corncob formulation and drinking water, which is similar to the colonization patterns under a natural condition where bacterial symbionts are acquired throughout feeding or drinking water. Our larval rearing experiments evidently showed negative effects of *E. cloacae*-GFPD on the development speed of *An. stephensi* larvae. These findings are in agreement with a previous study showed that *E. cloacae* could not isolate from the midgut of insectary specimens of *An. albimanus* Weidemann from southern Mexico whereas the bacteria was already isolated from field specimens (51). However, a great diversity of microbiota of larvae and adult mosquito gut has been already reported by other researchers (57, 45, and 15). However, it is currently unclear why the *E. cloacae* symbiont is not recycling in larval habitats at insectary condition.

In contrast to the larvae, colonization of the *E. cloacae*-GFP-D bacteria proceeds rapidly in adult mosquito midguts following sugar or blood meal. Colonization occurred within a few hours after taking meal and maximized 24h post blood meal. The blood proteins apparently caused quick growth of midgut bacteria and when blood digestion is completed (on day 6 or later) most bacteria were defeated with blood remains. The peak activity of *E. cloacae*-GFP-D would be synchronized with ookinete formation of *Plasmodium* parasite within the mosquito midguts. By proliferation of *E. cloacae-GFP-D* in the adult midgut, it could disrupt development of *Plasmodium* parasite directly by remarkable production of defensin, a killing parasite molecule, and indirectly by induction of *Anopheles* immune responses (33, 66, 67, 25). In this experiment, the bacteria were administrated via sugar bait, which is similar to the colonization patterns under a natural condition where bacterial symbionts are acquired throughout sap feeding.

Adult mosquitoes may acquire bacteria from at least one of the three following sources: animal host skin while taking blood meal, plant saps through sugar feeding, and transstadially transmitted bacteria from larvae to adult (46, 59, 68). Knowing bacterial acquisition routes is essential and play important role for paratransgenic approach against malaria vectors since it commands how to introduce transgenic bacteria to the field condition. We found that *E. cloacae*-GFP-D is not able to propagate in the larval habitat and midgut and it does not transfer transstadially from larvae to pupae as well as not from pupae to adults, so the mosquitoes are not able to take up the bacteria from larval habitats. This indicates that introduction of *E. cloacae*-GFP-D to breeding sites of *An. stephensi* is not recommended in a paratransgenic approach. In contrast, results of introduction of the bacteria via sugar bait containing modified bacteria were promising. Propagation of the bacteria in the adult midgut and it’s prolonged stability in sugar bait revealed that sugar bait is an effective mean to introduce engineered bacteria into field mosquito populations. This may be achieved by placing sugar bait stations with attractive material such as fruit juice embedded at attractive places such as pit shelters, black box trap, and earthenware crock close to breeding sites of *Anopheles* mosquitoes (53, 67, 69). Sugar bait station was evaluated in semi-field condition by Mancini et al. (70) and showed that the modified bacteria were effectively capable of spreading at high rate in different *An. stephensi* and *An. gambiae* populations, and successfully colonizing in the mosquito midguts. Recently Kotnis and Kuri (71) evaluated scenarios to calculate a number of required sugar baits and bait distribution to prevent a malaria outbreak. In our experiment, the *E. cloacae*-GFP-D was stable well in the cotton pads and remained viable at high rate through 11d which support suitability of sugar bait as a worthy mean for bacterial introduction to field. However, the stability and survival of *E. cloacae*-GFP-D in sugar bait should be evaluated in semi-field and field conditions in advance to use it in a real paratransgenic strategy.

The *E. cloacae* strain we used was easily cultivated outside the insect host on normal and cheap microbiological media and also genetically manipulated. In this study, we successfully used a GFP-defensin recombinant strain of the *E. cloacae* symbiont which allowed tracing of cells in plates originated from specimens such sugar baits and dissected midgut of larvae and adult mosquitoes. This bacterium was orally acquired successfully by adult mosquitoes from sugar bait that can be easily administered under semi-field and field conditions. *Enterobacter cloacae* have already been tested to deliver, express, and spread foreign genes in termite colonies (72) and mulberry pyralid moth, *Glyphodes pyloalis* (73).

The corncob formulation we prepared in this study was floated satisfactory on water surface for two days to supply the modified bacteria in insectary condition. This formulation was simple and needs to be developed to other known formulation such granule. Corn-cob has been successfully used as granule to supply *Bacillus thuringiensis* and *B. sphaericus* to control various larval mosquitoes in semi-field and field conditions (74–76). Factors potentially may influence the efficiency of formulation such as dosage of formulation, precipitation, flooding of the treated sites, and presence of other aquatic animals like fishes should be tested.

## Conclusion

Sugar bait station is the best method for introduction of *E. cloacae*-GFP-D into the field condition. The population of the bacteria was increased dramatically within 24h post blood meal. It can interrupt malaria parasite development in the mosquito midgut. This administration is similar to the colonization patterns under a natural condition where bacterial symbionts are acquired throughout sap feeding. On the other hand, lack of proliferation of the bacteria in breeding sites and subsequently in the larval midgut disapproved introduction of the bacteria in *Anopheles* larval habitat for a paratransgenetic approach.
